# Glabridin Alleviates Oxidative Stress-Induced Osteoporosis by Targeting the Akt/NF-ĸB and Akt/GSK-3β Pathways

**DOI:** 10.3390/ijms26072949

**Published:** 2025-03-24

**Authors:** Chittipong Tipbunjong, Wipapan Khimmaktong, Tanaporn Hengpratom, Thanvarin Thitiphatphuvanon, Chumpol Pholpramool, Piyaporn Surinlert

**Affiliations:** 1 Division of Health and Applied Sciences, Faculty of Science, Prince of Songkla University, Songkhla 90110, Thailand; chittipong.t@psu.ac.th (C.T.); wipapan.k@psu.ac.th (W.K.); tanaporn.he@psu.ac.th (T.H.); 2 Faculty of Medicine, Kasetsart University, Bangkok 10900, Thailand; thanvarin.t@ku.th; 3 Department of Physiology, Faculty of Science, Mahidol University, Bangkok 10400, Thailand; chumpol.pho@mahidol.ac.th; 4 Thammasat University Research Unit in Synthesis and Applications of Graphene, Thammasat University, Pathum-Thani 12120, Thailand; 5 Chulabhorn International College of Medicine, Thammasat University, Pathum-Thani 12120, Thailand

**Keywords:** antioxidant, bone, diabetes, glabridin, hyperglycemia, osteoporosis

## Abstract

Diabetes-related osteoporosis has been known to be a consequence of oxidative stress caused by excessive reactive oxygen species (ROS) production in the tissues. Despite the increase in the number of individuals with diabetes-related osteoporosis year on year, there is still no effective drug that does not induce adverse side effects. Glabridin, which exerts hypoglycemic effects and possesses antioxidant properties, may have beneficial effects in the treatment of diabetes-related osteoporosis. In this study, we aimed to investigate the preventive effects of glabridin in counteracting oxidative stress-induced bone loss and its underlying mechanisms. A diabetic rat model was established by a single intraperitoneal injection of streptozotocin into male Wistar rats. The diabetic rats were orally gavaged daily with glabridin or glyburide for 8 weeks. The presence of diabetes significantly decreased the rats’ tibia length, bone thickness, epiphyseal plate length, and collagen deposition compared to the control rats; in comparison, treatment with glabridin for 8 weeks significantly reversed these effects. In our in vitro study, the treatment of MC3T3-E1 preosteoblasts with glabridin up to 7.5 µM for 48 h showed no cytotoxic effect. However, pretreatment with glabridin significantly prevented oxidative stress-induced inhibition of cell proliferation. In addition, glabridin significantly diminished ROS production, restored antioxidant enzyme activity, and mitigated cellular apoptosis. These effects occurred by stimulating the phosphorylation of Akt, GSK-3β, and P65 NF-ĸB proteins. The above results show that glabridin alleviated oxidative stress-induced bone loss and osteoblast cell apoptosis by modulating the expression of the Akt/NF-ĸB and Akt/GSK-3β pathways.

## 1. Introduction

Diabetes mellitus (DM) is a metabolic disorder characterized by an increase in blood glucose levels. The International Diabetes Federation has reported an increase in the prevalence of diabetes globally over the past few decades, reporting that it will reach 629 million cases by the year 2045 [1]. Individuals with diabetes suffer from a number of health complications, including osteoporosis [2]. The results of previous studies have highlighted the high incidence of osteoporosis among individuals with diabetes [2], characterized by a decrease in bone mass and bone density that normally occurs in older people. Diabetic individuals with osteoporosis suffer from pain and an increased risk of bone fracture, both of which have an adverse impact on quality of life. The hyperglycemic condition has been reported to cause osteoporosis through the inhibition of osteoblast proliferation and differentiation via Akt/β-catenin signaling molecules [3]. Moreover, hyperglycemia can promote osteoblast apoptosis and autophagy through the production of reactive oxygen species [3,4].

**Figure 1:**
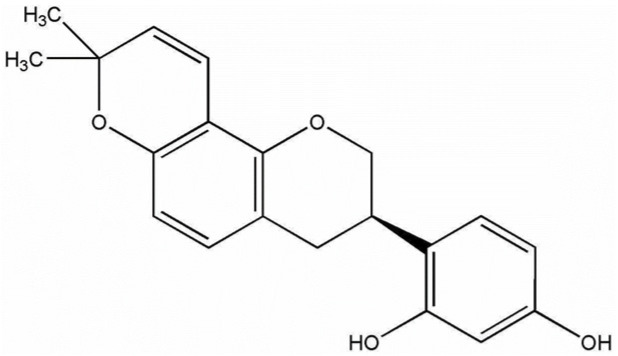
The chemical structure of glabridin.

Although the number of individuals with diabetes-related osteoporosis is increasing year on year, the common drugs on the market remain insulin, bisphosphonates, and raloxifene, the use of which has been reported to induce adverse side effects [5,6]. This limitation has, therefore, prompted researchers to explore new drugs and supplements for the treatment of this condition. Glabridin, a naturally occurring isoflavone compound, is the major active flavonoid in licorice. Its structure (Figure 1) consists of aromatic rings and hydroxy groups, which contribute to its beneficial antioxidant properties [7]. The underlying mechanisms of antioxidants from derived natural compounds have been reported to protect cells from ROS accumulation through the direct scavenging of free radicals [8], enhanced antioxidant enzyme expression [9], and the regulation of oxidative stress-responsive genes [10]. In congruence, glabridin has been reported to demonstrate the ability to directly scavenge free radicals [10]. In addition, it has been reported to enhance the expression of antioxidant enzymes such as superoxide dismutase (SOD) in brain tissue [11], SOD and catalase in monocytes [12], and SOD and glutathione peroxidase (GPx) in osteoblast cells [13], which lead to different endpoints that result in health benefits. Moreover, glabridin has been shown to stimulate Akt [14] and AMPK [15] expression while concurrently inhibiting RANKL and osteoclastogenesis expression [4] in bone cells, which are important signaling pathways for bone formation. It also increases the expression of ALP, OPN, OPG, OC, and BMPs [4] in osteoblast cells, which are vital for bone health and homeostasis. Glabridin has also been reported to exhibit hypoglycemic potential in diabetic rat models, with corresponding improvement in liver fibrosis [16], tendon inflammation [17], and nephropathy [18] caused by diabetes.

In light of the above finding, it is possible that glabridin may prevent bone loss and osteoblast apoptosis in individuals with diabetes. However, the protective effects of glabridin on oxidative stress-induced osteoporosis have yet to be reported. In this study, we aimed to elucidate the protective effects of glabridin against oxidative stress-induced bone loss and its underlying mechanisms.

## 2. Results

### 2.1. Glabridin Protected Against High Glucose-Induced Bone Loss in the Tibia of Rats

**Figure 2:**
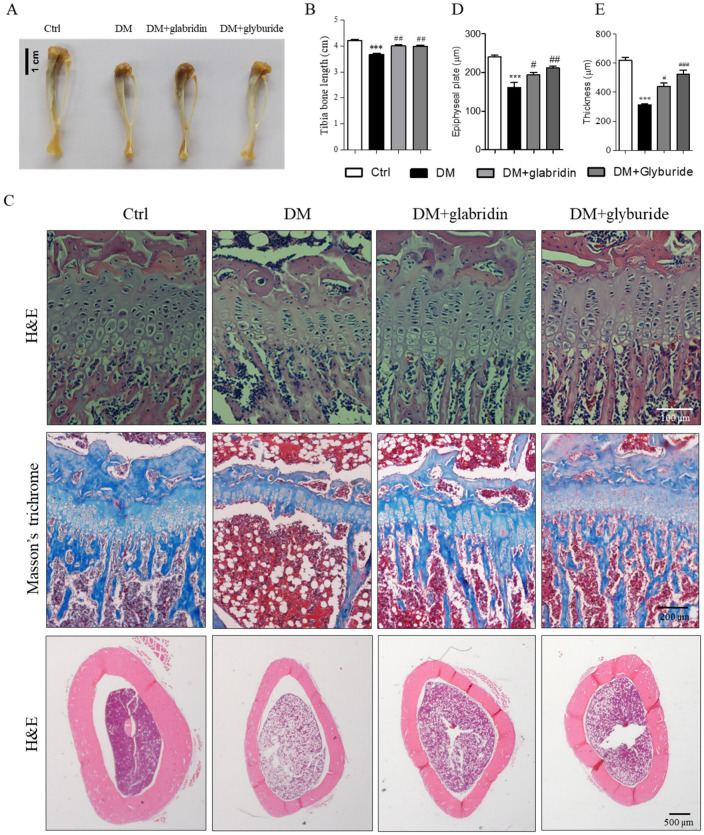
Effect of glabridin treatment on hyperglycemia-induced bone loss. Wistar rats were i.p. injected with normal saline (Ctrl) or streptozotocin (60 mg/kg) to induce diabetes mellitus (DM). The DM rats were treated with glabridin (DM + glabridin) or glyburide (DM + glyburide) for 8 weeks. Representative images of the tibia bone (**A**) and its length (**B**). Histology of the tibia stained with hematoxylin and eosin (H&E) and Masson’s trichrome (**C**). Length of the epiphyseal plate (**D**) and compact bone thickness (**E**). *** *p* < 0.001 when compared to the healthy control group; ^#^ *p* < 0.05, ^##^ *p* < 0.01, and ^###^ *p* < 0.001 when compared to the DM group.

After 8 weeks, the tibia length of rats in the DM group was significantly shorter than that of the rats in the Ctrl group. In contrast, the tibia length of rats in the DM + glabridin and DM + glyburide groups was significantly longer than that of the rats in the DM group (Figure 2A,B). In addition, bone histology revealed a significant decrease in the epiphyseal plate length in the DM group compared to the Ctrl group (Figure 2C,D). Both the zone of proliferation and the zone of hypertrophy were affected by the presence of diabetes. Interestingly, treatment with glabridin and glyburide after diabetic induction significantly reversed these effects. In addition, the amount of collagen deposition at the epiphyseal plate, which is important for bone growth, also significantly decreased in the DM group compared to the Ctrl group. However, treatment with glabridin and glyburide significantly restored the amount of collagen deposition (Figure 2C). Besides the epiphyseal plate, the bone thickness at the mid shaft of the tibia bone was found to be significantly thinner in the rats in the DM group compared to the rats in the Ctrl group. However, the bone thickness of the DM rats that received glabridin or glyburide daily for 8 weeks increased significantly compared to the rats in the DM group (Figure 2C,E).

### 2.2. Glabridin Attenuated Oxidative Stress-Induced Cell Cycle Arrest in MC3T3-E1 Preosteoblasts

**Figure 3:**
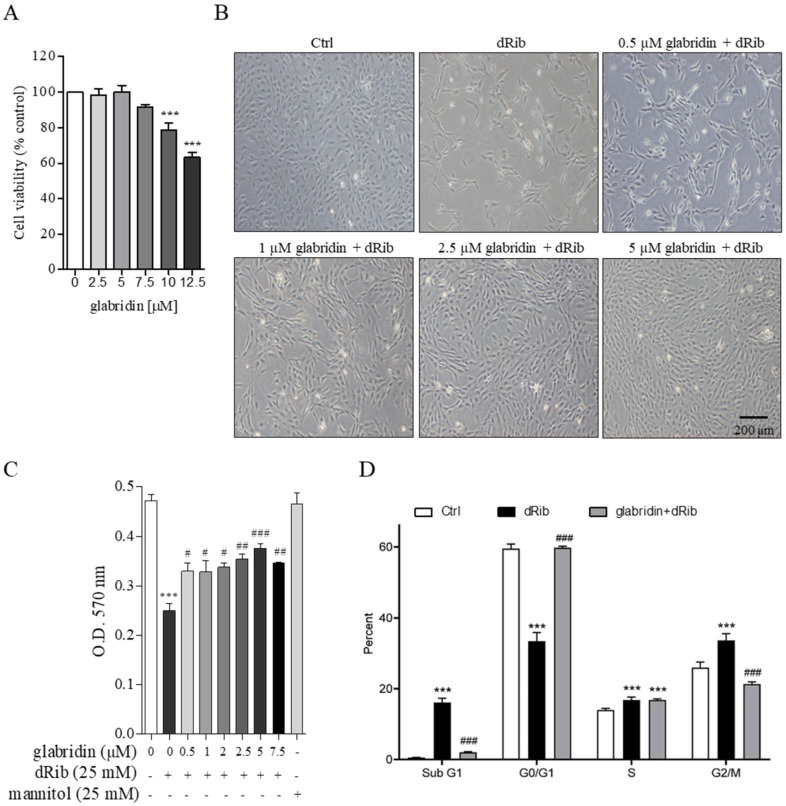
Effect of glabridin treatment on oxidative stress-induced cell cycle arrest. MC3T3-E1 preosteoblasts were treated with 2.5–12.5 µM glabridin for 48 h and cell viability was determined using an MTT assay (**A**). MC3T3-E1 preosteoblasts were pretreated with glabridin for 3 h before exposure to oxidative stress conditions (dRib). Representative images of the MC3T3-E1 preosteoblasts (**B**) and cell viability (**C**) after treatment in HG for 48 h. The percent of cell cycle distribution was determined using flow cytometry (**D**). *** *p* < 0.001 when compared to the Ctrl group; ^#^ *p* < 0.05, ^##^ *p* < 0.01, and ^###^ *p* < 0.01 when compared to the dRib group.

MC3T3-E1 is a preosteoblast cell line whose behavior is similar to osteoblasts and is a well-known model for in vitro bone research. To create oxidative stress conditions, preosteoblasts were incubated with 25 mM 2-deoxy-D-ribose (dRib). Treatment with glabridin up to 7.5 µM for 48 h showed no toxic effects on MC3T3-E1 preosteoblasts (Figure 3A). However, pretreatment with glabridin (1.0–7.5 µM) significantly prevented oxidative stress-induced cell proliferation inhibition (Figure 3B,C). The flow cytometry results revealed oxidative stress-induced preosteoblast cell cycle arrest at the sub-G1, S, and G2/M phases. However, pretreatment with 5 µM glabridin significantly attenuated this effect (Figure 3D).

### 2.3. Glabridin Prevented 2-Deoxy-D-ribose-Induced ROS Production and Apoptosis

**Figure 4:**
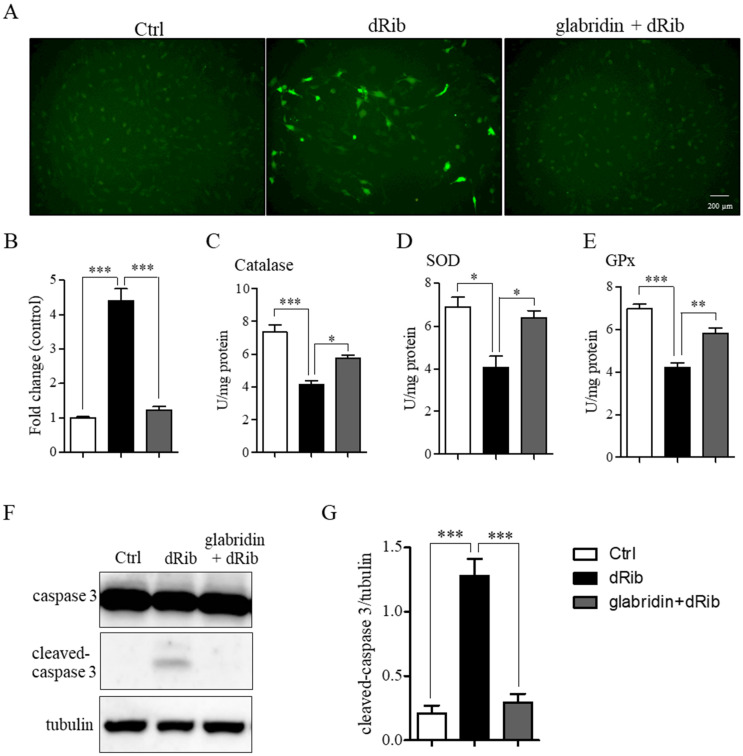
Effect of glabridin pretreatment on 2-deoxy-D-ribose-induced ROS production and apoptosis. MC3T3-E1 preosteoblasts were pretreated with glabridin for 3 h before exposure to oxidative stress conditions (dRib) for 48 h. Representative images of ROS production (**A**) and fluorescence intensity (**B**). Antioxidant enzyme activity: catalase (**C**), SOD (**D**), and GPx (**E**). Representative images of apoptosis protein (**F**) and band intensity (**G**). Data are shown as means ± SEM. * *p* < 0.05, ** *p* < 0.01, and *** *p* < 0.001 compared to the corresponding group.

In oxidative stress conditions, MC3T3-E1 preosteoblasts showed a significant increase in ROS production with a concurrent decrease in antioxidant enzyme activity, CAT, SOD, and GPx compared to the control. Pretreatment with glabridin significantly diminished ROS production (Figure 4A,B) and restored antioxidant enzyme activity (Figure 4C–E). The increase in ROS production combined with the decrease in antioxidant enzyme activity led to cellular apoptosis, confirmed by significant increases in cleaved caspase-3 levels. However, pretreatment with glabridin significantly mitigated these effects (Figure 4F,G).

### 2.4. Glabridin Targets the Akt/NF-ĸB and Akt/GSK-3β Pathways in MC3T3-E1 Preosteoblasts in Oxidative Stress Conditions

**Figure 5:**
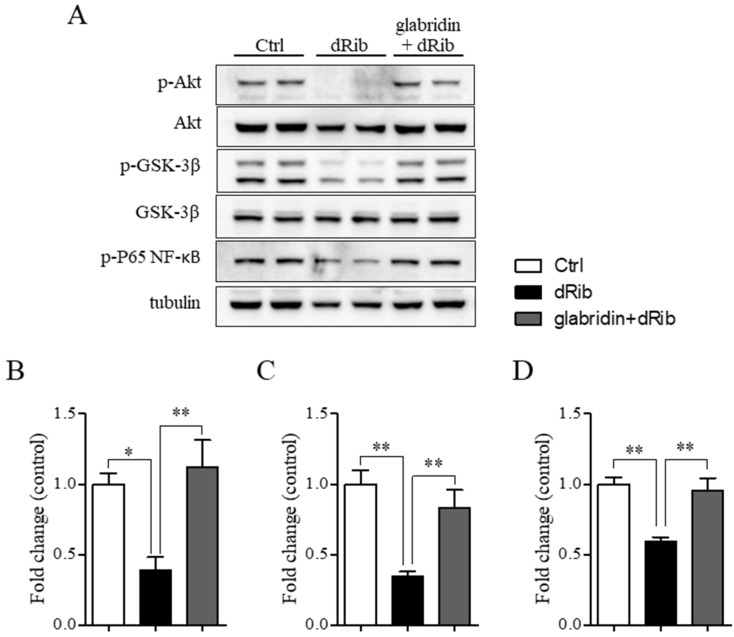
The underlying mechanism of glabridin prevents oxidative stress-induced MC3T3-E1 preosteoblast apoptosis. MC3T3-E1 preosteoblasts were pretreated with glabridin for 3 h before exposure to oxidative stress conditions (dRib) for 48 h. Representative images of Western blotting (**A**) and band intensity of p-Akt (**B**), p-GSK-3β (**C**), and p-P65 NF-κB (**D**). Data are shown as means ± SEM. * *p* < 0.05 and ** *p* < 0.01 compared to the corresponding group.

**Figure 6:**
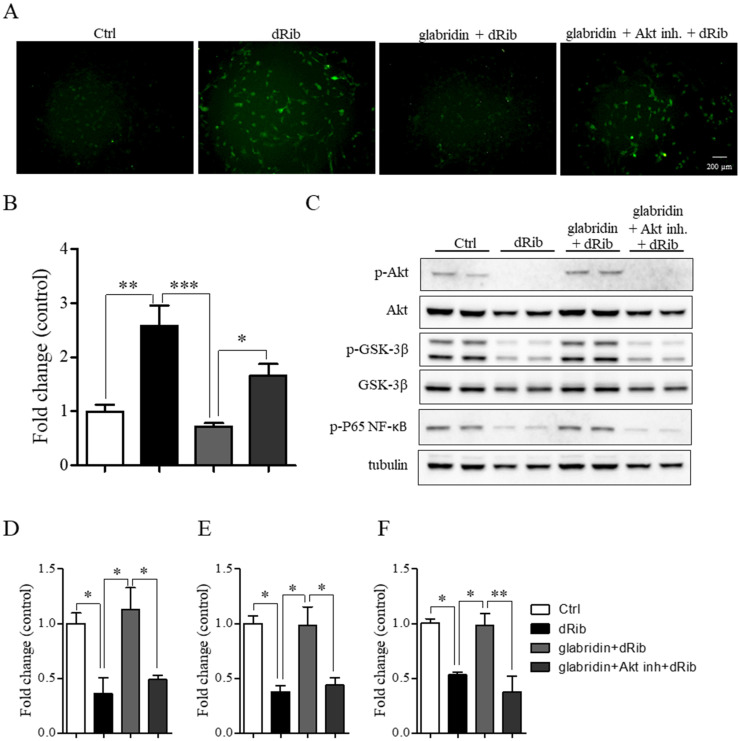
Phosphorylation of GSK-3β and P65 NF-ĸB took place downstream of phosphorylated Akt in glabridin to prevent oxidative stress-induced preosteoblast apoptosis. MC3T3-E1 preosteoblasts were pretreated with glabridin with or without the Akt inhibitor for 3 h before exposure to oxidative stress conditions (dRib) for 48 h. Representative images of ROS production were obtained using an H_2_DCFDA assay (**A**) and the fluorescence intensity was measured (**B**). Representative images of Western blotting (**C**) and quantification of band intensity for p-Akt (**D**), p-GSK-3β (**E**), and p-P65 NF-κB (**F**). Data are shown as means ± SEM. * *p* < 0.05, ** *p* < 0.01, and *** *p* < 0.001 compared to the corresponding group.

In oxidative stress conditions, the phosphorylation of Akt, GSK-3β, and P65 NF-ĸB proteins significantly decreased compared to the healthy control group. However, pretreatment with glabridin before exposure to oxidative stress conditions for 48 h significantly increased the phosphorylation of these proteins compared to the hyperglycemic group (Figure 5A–D). To ascertain whether the increase in the phosphorylation of Akt associated with glabridin prevents oxidative stress-induced preosteoblast apoptosis, Akt inhibitor IV, which inhibits Akt phosphorylation and activation, was applied. Interestingly, blockade with the 1 µM Akt inhibitor significantly eradicated the protective effect of glabridin against 2-deoxy-D-ribose-induced ROS production (Figure 6A,B). In addition, the level of phosphorylated GSK-3β and P65 NF-ĸB increased **(**Figure 6C,D). These data confirm that the phosphorylation of GSK-3β and P65 NF-ĸB took place downstream of phosphorylated Akt in glabridin to prevent oxidative stress-induced preosteoblast apoptosis.

## 3. Discussion

Hyperglycemic rats have been reported to experience a decrease in bone maximum load and energy absorption, possess a low number of osteocytes, and exhibit increased porosity and mineralization in cortical bone and decreased bone mass [19,20]. Glabridin has been reported to possess hypoglycemic properties able to ameliorate the deleterious effects caused by diabetes, including liver fibrosis [16], tendon inflammation [17], and diabetic nephropathy [18]. In addition, the metabolic parameters, including blood sugar level and liver function, of DM rat models significantly improved after receiving glabridin for 8 weeks compared to the beginning of treatment [16]. This hypoglycemic effect induced by glabridin has been proposed, at least in part, to be related to its antioxidant properties [21]. However, further research on the underlying hypoglycemic mechanism of glabridin is required. Apart from this hypoglycemic effect, the phytoestrogen naringenin has been reported to protect against diabetes-related osteoporosis by modulating osteogenesis, osteoclastogenesis, and macrophage polarization by stimulating the expressions of Runx-2 and osteocalcin proteins [22,23]. In addition, the phytoestrogen genistein has also been reported to prevent diabetes-related bone loss by stimulating the β-catenin/Runx-2 pathway, leading to the expression of bone protector osteoprotegerin protein [24]. In light of these findings, glabridin, which also exhibits estrogenic activity [25] and has also been reported to modulate β-catenin, Runx-2, and osteocalcin expression [15,26], could prevent hyperglycemia-induced bone loss through the same mechanism as naringenin and genistein.

Indeed, we have shown in the present study that glabridin could prevent osteoporosis induced by hyperglycemia in rats. We found that glabridin reversed the effects of streptozotocin on tibia length and histology reflective of osteoporosis in rats. This effect is similar to that of the positive control, glyburide. It has been shown in previous studies that hyperglycemia causes osteoporosis through an imbalance between osteoclast and osteoblast function [27], which occurs due to an imbalance between ROS production and antioxidant defense [21]. Given that glabridin exerted antioxidant properties both through direct scavenging of ROS and enhanced intracellular antioxidant enzyme activity in osteoblasts, it may attenuate the pathogenesis of hyperglycemia and its impact on bone length and histology. In addition, it has been reported that natural extracts exert hypoglycemic effects by increasing insulin levels and downregulating osteoclastogenesis [28], processes which, in turn, prevent diabetes-induced osteoporosis. These results are consistent with those of other reports noting that natural extracts that possess antioxidant properties can attenuate hyperglycemia-induced bone loss [28,29].

In order to gain better insight into the cellular mechanism of glabridin, we chose MC3T3-E1 preosteoblasts, whose behavior and physiological response are similar to osteoblasts, as a model for further examination. Our results showed that oxidative stress caused by 2-deoxy-D-ribose treatment induced MC3T3-E1 preosteoblast cell cycle arrest at the sub-G1, S, and G2/M phases. Oxidative stress has been reported to induce C2C12 myoblast cell cycle arrest at the sub-G1 and G2/M phases by modulating Sirt-1 and Sirt-2 gene expression [30] and aberrant cell proliferation by mediating protein O-GlcNAc modification [31]. However, these effects were prevented by glabridin pretreatment. Even though glabridin has been reported to cause cancer cell cycle arrest and proliferation inhibition by downregulating the Braf/MEK [32] and JNK1/2 signaling pathways [33], in this study, glabridin prevented the MC3T3-E1 preosteoblast cell cycle arrest and proliferation inhibition induced by oxidative stress. This effect is exerted via the estrogenic activities of glabridin. MC3T3-E1 preosteoblast cells have been reported to express both estrogen receptor-α and β [33], in addition to the membrane estrogen-specific G protein-coupled receptor 30 (GPR30/GPER) [34]. Estrogenic activity has been reported to induce cell proliferation by stimulating progression through the G1 phase of the cell cycle by activating Cdk4 and Cdk2 proteins [35]. In addition, it also regulates the expression and function of c-Myc and cyclin D1, which are crucial for cell cycle progression [36].

Mitochondria are the major source of endogenous ROS production in living organisms. However, under normal cellular physiology, the rate of ROS production and scavenging are balanced to protect cells from ROS accumulation that eventually leads to apoptosis [37]. The accumulation of ROS caused by high glucose has been reported to inhibit osteoblast proliferation and differentiation by inducing pyroptosis [3,38], promote mitochondrial perturbation [38], and modulate gene expression [39] in vitro, which disrupt bone growth, development, and homeostasis. In addition, ROS accumulation impairs the bone microstructure by decreasing bone volume, disrupting bone trabecula structures, and reducing the thickness and strength of cortical bone in rats [40]. Several methods have been reported to successfully inhibit ROS production; for example, increasing antioxidant levels can inhibit the release of LC3 autophagy molecules [41], inhibit the transmembrane NADPH oxidase enzyme [42], and increase antioxidant enzyme activity [43].

Cellular reactive oxygen species (ROS) are the derivatives of molecular oxygen molecules that impact intracellular balance. The excessive production of ROS leads to cell cycle arrest, inhibition of proliferation and differentiation, and cellular apoptosis [44]. High glucose has been reported to be a cause of excessive ROS production through the activation of the p38/JNK/cytochrome c and/or ERK/Bad pathways, leading to increases in cleaved caspase-8 and cleaved caspase-3 levels, which are the key mediators of apoptosis [30,45]. The chemical structure of glabridin contains dihydroxyl groups on the benzene ring, which has special antioxidant properties and can directly interact with ROS molecules and neutralize them [46]. In addition, glabridin has been reported to be a phytoestrogen that can bind with estrogen receptors and mediate the signaling cascade, leading to increased antioxidant activity (SOD, CAT, and GPx) [21]. Moreover, it has been reported to modulate the expression of the p53/Bcl-2/PARP signaling pathway, which eventually leads to anti-apoptosis properties [47]. Both the chemical and cellular antioxidant activity of glabridin can prevent ROS production and accumulation in hyperglycemic conditions, which, in turn, decreases the levels of cleaved caspase-8 and cleaved caspase-3 and mitigates cell apoptosis.

Although the authors of previous studies have reported the systemic metabolic improvement of DM rat models after receiving glabridin [16,17], our in vitro results provide evidence to support the direct effect of glabridin on preosteoblast cells under oxidative stress conditions via the Akt/NF-κB and Akt/GSK-3β signaling pathways, leading to cell proliferation stimulation and apoptosis prevention. As a phytoestrogen, binding with estrogen receptors can mediate diverse signaling cascades, leading to different endpoints. Glabridin has been reported to bind with estrogen receptors, particularly ER-α [25,48], and modulate the activity of PI3K/Akt in osteoblast cells in hyperglycemic conditions [14]. Akt activation under hyperglycemic conditions can protect osteoblast cells from cell death, apoptosis, cell cycle arrest, and bone loss [14,49]. Several underlying Akt signaling molecules have been reported in the literature. Among them, NF-ĸB and GSK-3β activities are reported to be modulated under hyperglycemic conditions [50,51]. The activation of NF-κB depends on the phosphorylation and degradation of IκBα, which, in turn, leads to the translocation of the p65/p50 subunit to the nucleus [52]. By binding to the DNA consensus sequence, it can modulate the anti-apoptotic factors Fas, BCL-2, Caspase, and Survivin [53]. Conversely, the inactivation of GSK-3β through phosphorylation has been reported to prevent apoptosis [54] and induce the cell proliferation of human β-cells [55]. In addition, the phosphorylation of GSK-3β also enhances bone formation via the Akt/GSK-3β pathway [56]. Besides Akt/NF-ĸB and Akt/GSK-3β, other signaling molecules including p38 MAPK, JNK1/2, and Wnt/β-catenin have been reported to be modulated by glabridin and may be involved in diabetes-related osteoporosis [57]. Given that we only focused on the Akt/NF-ĸB and Akt/GSK-3β pathways in this study, further research into glabridin’s other mechanisms of action in preventing diabetes-related osteoporosis is needed. The relevant biological mechanisms, in particular, the p38 MAPK/Nrf2/NF-κB, p38 MAPK, JNK1/2, and Wnt/β-catenin pathways, are of significant interest. Moreover, interplay and cross-talk between pathways are also possible.

## 4. Materials and Methods

### 4.1. Animal Ethics Approval

The animal handling protocol and experimental design used in this study were conducted in accordance with and approved by the Animal Ethics Committee of Prince of Songkla University, Songkhla, Thailand (MOE 0521.11/095, Ref.5/2018).

### 4.2. Animals and Experimental Procedures

The 8-week-old male Wistar rats (200–250 g) were cared for under constant temperature (23 ± 2 °C) and humidity (50 ± 10%) conditions with a 12 h light–dark cycle for one week prior to the experiments. The diabetic rat model was established by a single intraperitoneal injection of freshly prepared streptozotocin (60 mg/kg) to overnight fasted animals, and DM status was validated based on the rats’ blood glucose levels, which showed levels higher than 250 mg/dL 72 h after injection. The control rats were injected with normal saline. The rats were divided into 4 groups, namely the healthy control (Ctrl), diabetic control (DM), diabetic rats receiving 40 mg/kg/d glabridin for 8 weeks (DM + glabridin), and diabetic rats receiving 5 mg/kg glyburide (positive control) for 8 weeks (DM + glyburide) groups.

### 4.3. Histological Study

After 8 weeks of treatment, the rats were euthanized and their tibia bones were carefully excised. Tibia bone length was measured before fixation in 10% formalin overnight before being subjected to decalcification, dehydration, and finally paraffin embedding. The bone tissues were sectioned at 5 μm with a rotary microtome. The tissues were further stained with hematoxylin–eosin (H&E) and Masson’s trichrome following the standard protocol.

### 4.4. Cell Culture and Treatment

The mouse preosteoblastic cell line (MC3T3-E1) was purchased from the American Type Culture Collection (ATCC). The MC3T3-E1 cells were maintained in alpha-minimum essential medium (α-MEM) containing 10% fetal bovine serum and 1% penicillin–streptomycin at 37 °C in a humidified CO_2_ incubator. The cells were subcultured once they reached 80% confluence. To perform the experiments, the MC3T3-E1 cells were seeded into a 6-well plate overnight to enable cell attachment and growth. The cells were pretreated with or without Akt inhibitor IV and 5 μM glabridin for 3 h prior to incubation under oxidative stress conditions with 25 mM 2-deoxy-D-ribose (dRib). The treated cells were further cultured for 48 h before another experiment was performed.

### 4.5. Cell Viability Assay

The MC3T3-E1 cells were subcultured and seeded into a 96-well plate overnight to enable cell attachment and growth. The cells were then cultured in media containing glabridin ranging from 2.5 to 12.5 μM for 48 h. Thereafter, the treated cells were transferred to a growth medium containing 0.5 mg/mL MTT for another 3 h. After incubation, the MTT solution was discarded and replaced with DMSO. After constant agitation, the absorbance of the formazan solution was analyzed at 570 nm.

### 4.6. Flow Cytometry

After treatment, the MC3T3-E1 cells were trypsinized and immediately fixed in 70% ethanol overnight. After several washes with PBS, the cells were incubated with RNase A at 37 °C for 15 min. After incubation, propidium iodide (PI) was directly added to the tube before being subjected to flow cytometry using a BD FACSCanto™ flow cytometer (BD Biosciences, Milpitas, CA, USA).

### 4.7. H_2_DCFDA Assay

After treatment, the cells were washed with PBS and incubated in media containing 10 µM 2′,7′-dichlorodihydrofluorescein diacetate (H_2_DCFDA) for 30 min. The treated cells were washed with PBS and the nuclei were stained with Hoechst 33342. Images of the ROS were then taken using a fluorescence microscope and their intensity was analyzed using the ImageJ software (version 1.8.0).

### 4.8. Antioxidant Enzyme Activity Assay

After treatment, the cells were collected via trypsinization and subjected to protein extraction. The protein concentration was determined using the BCA assay kit. Antioxidant enzyme activity was analyzed using the enzyme activity assay kits (Elab Science, Houston, TX, USA) and presented as U/mg protein.

### 4.9. Western Blotting

Briefly, the protein was extracted using RIPA buffer containing protease and phosphatase inhibitors and separated via SDS-PAGE before being transferred to a PVDF membrane. The membrane was incubated with 5% goat serum in Tris-buffered saline (TBS) for 1 h at room temperature to prevent non-specific binding. Thereafter, it was incubated with a primary antibody diluted in 5% goat serum in TBS overnight at 4 °C. After washing, the membrane was incubated with an HRP-conjugated secondary antibody. The membrane was examined with the enhanced chemiluminescence substrate and detected using the gel documentation imaging system. The protein band intensity was measured with the ImageJ software.

### 4.10. Statistical Analysis

Data were collected from at least 3 independent experiments and are presented as the mean ± standard error of the mean (SEM). The statistical analysis was conducted with a one-way analysis of variance followed by Tukey’s post hoc test. A *p*-value less than 0.05 was considered significantly different.

## 5. Conclusions

This study is the first report to evaluate the protective effect of glabridin against hyperglycemia-induced bone loss in the tibia of rats. Our results revealed that glabridin prevented bone loss by preventing osteoblast cell cycle arrest, diminished ROS production and accumulation, increased antioxidant enzyme activity, and inhibited cellular apoptosis. Moreover, our results also showed that glabridin alleviated hyperglycemia-induced bone loss and osteoblast cell apoptosis by modulating the expression of the Akt/NF-ĸB and Akt/GSK-3β pathways.

## Data Availability

Data are contained within the article and will be made available upon request.

## Abbreviations

The following abbreviations are used in this manuscript:

NF-κB	nuclear factor kappa B
GSK-3β	Glycogen synthase kinase-3 beta
Akt	protein kinase B
SOD	superoxide dismutase
GPx	glutathione peroxidase
CAT	catalase
ROS	reactive oxygen species
DM	diabetes mellitus
RANKL	receptor activator of nuclear factor kappa beta ligand
ALP	Alkaline phosphatase
BMPs	bone morphogenetic proteins
OPN	Osteopontin
OPG	osteoprotegerin
OC	osteocalcin
